# 3D Printing of Calcium Phosphate/Calcium Sulfate with Alginate/Cellulose-Based Scaffolds for Bone Regeneration: Multilayer Fabrication and Characterization

**DOI:** 10.3390/jfb13020047

**Published:** 2022-04-25

**Authors:** Nattanan Wattanaanek, Srisurang Suttapreyasri, Bancha Samruajbenjakun

**Affiliations:** 1 Orthodontic Section, Department of Preventive Dentistry, Faculty of Dentistry, Prince of Songkla University, Hat Yai 90112, Songkhla, Thailand; wa_n30@hotmail.com; 2 Department of Oral and Maxillofacial Surgery, Faculty of Dentistry, Prince of Songkla University, Hat Yai 90112, Songkhla, Thailand; srisurang.s@psu.ac.th

**Keywords:** bone regeneration, 3D printing, scaffold, calcium phosphate, calcium sulfate, alginate, cellulose

## Abstract

Congenital abnormalities, trauma, and disease result in significant demands for bone replacement in the craniofacial region and across the body. Tetra-compositions of organic and inorganic scaffolds could provide advantages for bone regeneration. This research aimed to fabricate and characterize amorphous calcium phosphate (ACP)/calcium sulfate hemihydrate (CSH) with alginate/cellulose composite scaffolds using 3D printing. Alginate/cellulose gels were incorporated with 0%, 13%, 15%, 18%, 20%, and 23% ACP/CSH using the one-pot process to improve morphological, physiochemical, mechanical, and biological properties. SEM displayed multi-staggered filament layers with mean pore sizes from 298 to 377 μm. A profilometer revealed mean surface roughness values from 43 to 62 nm that were not statistically different. A universal test machine displayed the highest compressive strength and modulus with a statistical significance in the 20% CP/CS group. FTIR spectroscopy showed peaks in carbonate, phosphate, and sulfate groups that increased as more ACP/CSH was added. Zero percent of ACP/CSH showed the highest swelling and lowest remaining weight after degradation. The 23% ACP/CSH groups cracked after 60 days. In vitro biocompatibility testing used the mouse osteoblast-like cell line MC3T3-E1. The 18% and 20% ACP/CSH groups showed the highest cell proliferation on days five and seven. The 20% ACP/CSH was most suitable for bone cell regeneration.

## 1. Introduction

Scaffold materials for tissue engineering are fabricated to closely resemble the physiological environment and the geometrical and physical characteristics of the desired tissue [1,2]. The biological features of seeded cells, as well as their survival, motility, proliferation, and metabolism, are all influenced by the scaffold material. As a result, it is an important component in bone tissue engineering [3].

Three-dimensional (3D) printing technologies used to fabricate scaffolds include stereolithography (SLA) [4], selective laser sintering [5], and fused deposition modeling (FDM) [6]. The printing of 3D scaffolds encourages cell accumulation, outgrowth, and differentiation for bone tissue regeneration [7]. They also establish a suitable 3D geometric pattern for living cells [8]. Moreover, these 3D printing technologies can easily form macro and microporous scaffolds to allow adequate nutrient supply and structural stability for the bio-environment. 3D printing can create the proper pore size and interconnected porosity of a scaffold by controlled programming and extrusion pressure factors. Direct ink writing is a printing technology for soft materials with computer aided control to fabricate a structure with low harm to cells, and it does not require additional techniques for a post-process. For example, SLA needs a monomer to complete the polymerization after printing [4] and requires removal of powders [5]. In addition, direct ink writing is capable of multi-stack manufacturing and combining multi-materials [9].

Many studies have used inorganic and organic components to fabricate scaffolds because they are the main components of bone [10]. Since an ideal material should match both the components and the structure of natural bone, the bioceramics group, which includes calcium phosphate (CP), is a very desirable basic material [11,12]. CP is the principle inorganic component of biological hard tissues such as bone and teeth. CP has good biocompatibility for biomedical applications, especially in the field of hard tissue regeneration [13]; however, the chemical and mechanical characteristics of the various phases differ [14]. Amorphous calcium phosphate (ACP) is a subclass of calcium phosphate formed with a chemical formula of Ca_3_(PO_4_)^2^·3H_2_O [15,16]. ACP is a glass-like structure with poorly arranged molecules positioned in the first stage of calcium and phosphate deposition in bone regeneration. This loose crystalline structure results in more bone deposit than hydroxyapatite [17]. Moreover, it was demonstrated that CP with alginate (Alg) could create 3D printed scaffolds with multiple layers and pores, which is appropriate for a bio-economic system [18]; however, after ACP transforms into hydroxyapatite, a residual graft remains in the defect longer than six months [19,20].

Calcium sulfate hemihydrate (CSH) has a long clinical history because of its quick setting qualities and strong biocompatibility [21,22,23]. CSH powder, for instance, transforms to calcium sulfate dihydrate (gypsum, CaSO_4_∙2H_2_O) when combined with water [24]; however, the in vivo CSH resorption rate of 1–2 months is far too fast [25]. One concept of an ideal synthetic material is to combine the characteristics of relatively slower ACP resorption with the rapid resorption of CSH at a ratio of 40:60. This was shown to have a resorption period of 3–6 months that closely mimicked the natural healing rate for new bone formation [26]. Increasing the amount of CSH into 3D printing significantly improved the compressive strength at days 7 and 14 [27].

Biocompatible natural organic polymer materials are good due to minimal irritation and toxicity after implantation in the human body [28]. Alg is a polymer often constructed as a scaffold that provides good chemical and physical characteristics for cell culture [26]. Since Alg is a polysaccharide, it influences the texture of the extracellular matrix causing tissue formation [29]. The interaction of Alg with divalent cations, such as calcium, causes alginate to gel causing poly-guluronic acid sequences chelated between the chains to dimerize [30]. Furthermore, a mixed gel allows for the construction of 3D scaffolds with the potential to manufacture scaffolds with custom-designed microarchitectures that mimic in vivo conditions [31]. The combination of Alg and cellulose is well documented due to its proper physical properties [32]. Moreover, a mixture of Alg and hydroxypropyl methylcellulose (HPMC) provided volume stability for 3D construction for 21 days, and human chondrocyte cells remained alive [33]. 

HPMC is one type of cellulose form that is a polysaccharide. Adding HPMC also promoted cell proliferation and cell functions [34], and HPMC is highly viscous [35]. Combining two polymers into an interpenetrated polymer network might be an excellent strategy to enhance the characteristics of these gels. Interpenetrated polymer networks were researched in the past, and they demonstrated improved mechanical qualities in some circumstances compared with single networks [36,37]. 

Previous research has not reported on mixtures of four components using 3D printing. This study aimed to determine the best combination of organic and inorganic substances using ACP/CSHand alginate/cellulose-based 3D printing for tissue engineering. The scaffolds were characterized, and the physicochemical and biological properties were evaluated.

## 2. Materials and Methods

### 2.1. ACP and CSH Preparation

ACP was prepared by adding 100 mL of 2.33 M sodium phosphate dibasic solution (Sigma-Aldrich, St. Louis, MO, USA) and 100 mL of 3.50 M calcium chloride (CaCl_2_) solution (Sigma-Aldrich, St. Louis, MO, USA) into sodium hydroxide that was dissolved in 100 mL of distilled water at pH 9 as a buffering medium. ACP was then filtered by high-power filtration and was freeze-dried for two days in accordance with a previous study [38]. CSH was purchased from Sigma-Aldrich, USA.

### 2.2. Printing Ink Preparation

The alginate/cellulose gel solution was prepared by dissolving 1 wt% Alg (Sigma-Aldrich, St. Louis, MO, USA) in a phosphate-buffered salt solution (PBS) at 120 °C. This method was modified from Eliaz et al. [32]. Then, 0.9 wt% HPMC (Sigma-Aldrich, St. Louis, MO, USA) was added to the solution as a water retention agent [33]. ACP/CSH was prepared to achieve a ratio of 2:3 before dispersion in the gels to obtain the printing ink. The printing ink was prepared for five experimental groups according to the ratio of ACP:CSH to the gel solution, which were designated as 13% CP/CS, 15% CP/CS, 18% CP/CS, 20% CP/CS, and 23% CP/CS. A 0% ACP/CSH was the control group. The printing ink suspension was filtered through a 110-μm filter to obtain a homogenous and desired particle size.

### 2.3. Scaffold Fabrication

**Figure 1:**
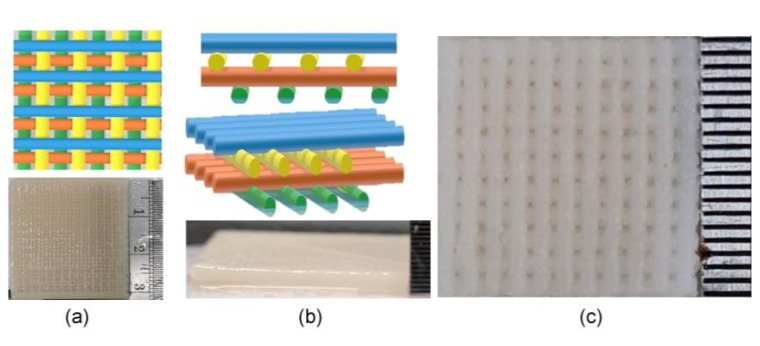
Filament patterns of the scaffolds. (**a**) Top view of the staggered filaments, (**b**) cross-section of staggered filaments, (**c**) scaffolds were cut after freeze-drying.

After gel formation for 24 h, the samples were delivered to a 3D printer (Bio X^TM^, CELLINK, Blacksburg, VA, USA). A 3 mL cartridge was installed with a 0.20 mm diameter needle for scaffold fabrication. The scaffold structures were created with 30 filaments/layer and a 1000-micron space between each filament using G-code generating software (Repetier-Host version 2.1.6, Willich, Germany). The scaffolds contained 10 layers. Each layer was staggered at 0° and 90° (Figure 1a–c). The printer was set at 45 mm/s, 350 kPa, and 25 °C. After every 2–3 layers, 0.1 mM CaCl_2_ was used as the cross-linking agent for structure fixation. The scaffold was then immediately immersed in the cross-linking agent for 60 min for a complete set and washed with distilled water five times. The scaffolds were separated into 10 × 10 × 2 mm^3^ and 7 × 7 × 2 mm^3^ sizes. All samples were freeze-dried for 3 h before sterilization with ethylene oxide gas for 3 h. Each scaffold group contained three samples.

### 2.4. Structural and Morphological Characterization

Scanning electron microscopy (SEM) (Quanta 400, Thermo Fischer Scientific, Brno, Czech Republic) was set at 15 kV and used to photograph the surfaces and cross-sectional aspects of the scaffolds. The SEM photos were also used to measure the pore size between each filament. The chemical compounds of all groups were confirmed by Fourier transform infrared (FTIR) spectra (VERTEX 70, Bruker, Rheinstetten, Germany) in the frequency range of 4000–400 cm^−1^.

### 2.5. Surface Roughness

Surface roughness of the scaffolds was measured by an optical profilometer (Flex, Nanosurf, Ecublens, Switzerland) and evaluated using dynamic force at a vibrating frequency of 165.871 kHz. The C3000 Control version 3.10.0 software presented the results.

### 2.6. Mechanical Characterization

Compressive strength was tested by a universal testing machine (Lloyd model LRX-Plus, Lloyd Instrument Ltd., London, UK). The scaffold was pressed onto a plate with a load cell of 0.5 kN at a rate of 0.5 mm/min and subjected to a strain of 80%.

### 2.7. Swelling Property

The samples were soaked in PBS solution and analyzed on days 1, 3, 5, and 7 at 37 °C. They were weighed after removing the excessive PBS. The swelling ratio was calculated using the following equation: Swelling ratio (%) = (Ws − Wd0)/Wd0 × 100, where Wd0 was the initial dry weight and Ws was the swollen weight.

### 2.8. Degradation Rate

All samples in each group were weighed (Wd) before incubation at 37 °C. The scaffolds were soaked in a PBS solution on days 1, 3, 7, 14, 21, 30, 60, and 90. On the days of measurement, the scaffolds were washed with distilled water three times before freeze-drying for 3 h. The remaining weight (Wr) was calculated. The percentage of remaining weight was calculated using the following equation: Wr (%) = (Wd0 − Wr)/Wd0 × 100, where Wd0 was the initial dry weight.

### 2.9. Cell Culture and Cell Seeding

The mouse osteoblast cell line MC3T3-E1 (ATCC, Manassas, VA, USA) was cultured with alpha-MEM medium (α-MEM, GibcoTM, Invitrogen, Carlsbad, CA, USA), 10% fetal bovine serum, 1% penicillin/streptomycin, and 0.1% Fungizone^®^. A 48-well plate was used for this experiment. The cells were seeded with 1 × 10^5^ cells/well, and the media were changed every three days with 500 mL/well.

### 2.10. Attachment, Viability, and Proliferation of Cells on the Scaffolds

SEM was used to observe the characteristics of osteoblast cell attachment on the scaffold surfaces on days 1 and 7. Viability of the cells on the scaffold surfaces was examined by fluorescence microscopy (ZEISS LSM 800, Carl Zeiss AG, Oberkochen, Germany). Fluorescein diacetate (FDA) was prepared by dissolving 5 mg/mL in acetone. FDA was dropped onto the seeded scaffolds after 24 h and 36 h of cell growth. The 48-well plate was incubated at 37 °C for 5 min [39] before nuclear staining, using 5 mg/mL of fluorescent mounting media (DAPI, Millipore-SigmaTM CalbiochemTM). After incubation for 5 min, the scaffolds were washed and blotted dry three to five times before observing cell viability.

PrestoBlue™ Cell Viability reagent (Invitrogen, Thermo Fischer Scientific, Waltham, MA, USA) was used to observe the living cells by the reduction of resazurin to highly fluorescent resorufin. After seeding the cells to 7 × 7 × 2 mm^3^ scaffolds, the scaffolds were moved to a new 48-well plate. The scaffolds were washed with PBS two times and incubated for 60 min at 37 °C prior to the test. The color changes were observed on days 1, 3, 5, and 7. The reagent was mixed with a fresh alpha-MEM medium at the ratio 1:10. The solution was then transferred to the scaffold. Cell proliferation was observed at a wavelength of 600 nm.

### 2.11. Statistical Analysis

The results are presented as mean ± standard deviation. Three independent analyses were performed for all experiments. The normal distribution of all data was tested using the Shapiro–Wilk test. One-way ANOVA and the Tukey HSD test were used to analyze the differences between groups. The statistical analysis used SPSS Statistics Bass 17.0 for Windows EDU (SPSS, Chicago, IL, USA). The statistical significance was set at *p* < 0.05.

## 3. Results

### 3.1. Scaffold Characterization

**Figure 2:**
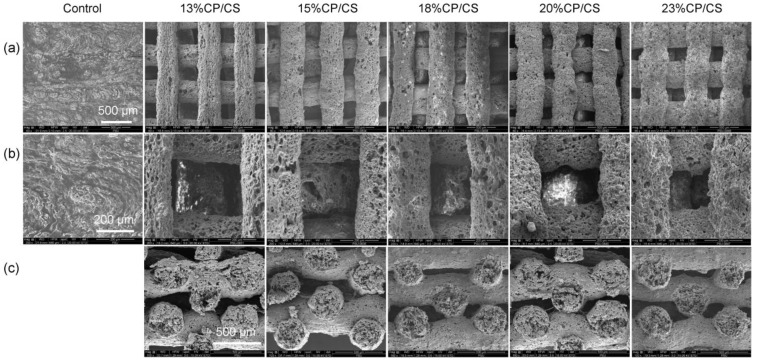
(**a**) SEM images of scaffold surfaces at 60× magnification, (**b**) scaffold surfaces at 200× magnification, (**c**) cross-section images at 200× magnification.

After completion of 3D printing, the filaments of the control group fused significantly, whereas the other groups did not (Figure 2a,b); therefore, pore size was not measured in this group. However, filament orientations of both the surfaces and cross-sections of the other groups are shown in Figure 2. From the top view at 60× magnification, the scaffolds presented smooth characteristics and a 0°/90° orientation of the filaments (Figure 2a). The staggered layers are illustrated in Figure 2c. At 200× magnification, the 15% CP/CS and 18% CP/CS groups had nearly the same mean pore size with an average of 299 ± 52 μm at the scaffold surface (Figure 2b). On the other hand, the 13% CP/CS, 20% CP/CS, and 23% CP/CS groups had higher mean pore sizes at the scaffold surface that averaged 312 ± 47 μm (Table 1).

The 15% CP/CS group had the largest cross-sectional mean pore size of 377 ± 69 μm. Nonetheless, the 13% CP/CS, 18% CP/CS, 20% CP/CS, and 23% CP/CS groups had an average cross-sectional pore size of 335 ± 78 μm with a significant difference (*p* = 0.000) between the control and five experimental groups. Nevertheless, no statistical differences were observed between any of the experimental groups (Table 1) (Figure 2c).

### 3.2. FTIR

The FTIR graph shows different peaks of each chemical compound. The stretching bands in the range of 566–1087 cm^−1^ indicated PO_4_^3−^ and the vibration of the P–O bond of the phosphate group was also at 1029 cm^−1^. Peaks of the carbonate groups appeared at 1467 cm^−1^ and 1552 cm^−1^. In addition, the peak between 500–1300 cm^−1^ indicated the presence of sulfate ions. The peak range of 594–1004 cm^−1^ was defined as SO_4_^2−^ and showed a very weak bond. The 23% CP/CS group showed more broad peaks than the other groups. The 18% CP/CS, 20% CP/CS, and 23% CP/CS groups showed broad vibration bands which indicated large amounts of the phosphate, carbonate, and sulfate groups from the CP/CS.

The peaks at 1740 cm^−1^ (carboxylic acids [–COOH]) and 1602–1642 cm^−1^ (carboxylate ions [–COO^−^]) were interpreted as alginate. The vibration line at 1052.2 cm^−1^ indicated the secondary alcohol group from cellulose.

**Figure 3:**
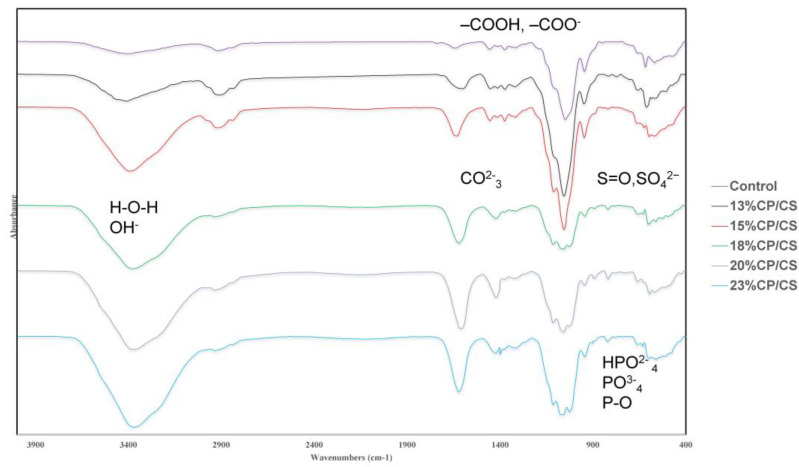
Comparison of FTIR graphs that show the chemical compositions of each compound, such as those in the hydroxyl, carbonate, phosphate, and sulfate groups.

Moreover, the vibration at 3308 cm^−1^ corresponded to the hydroxyl groups. The 3400 cm^−1^ area of the broad peak indicated O–H bonds from the water molecules. The amount of hydroxyl groups from alginate created a lower asymmetrical stretching band than the hydroxyl groups from CP, and it also showed a weaker molecular bond (Figure 3).

### 3.3. Surface Roughness

**Figure 4:**
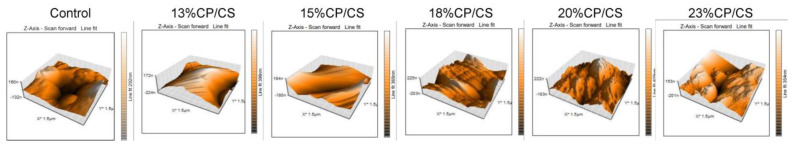
3D images of surface roughness.

The mean surface roughness average of the control group was 9 ± 4 nm, which was the smoothest. The highest mean surface roughness value was the 20% CP/CS group (62 ± 9 nm), whereas the 13% CP/CS, 15% CP/CS, 18% CP/CS, and 23% CP/CS groups showed an average mean surface roughness value of 50 ± 5 nm (Table 1); however, the control group was statistically different from the other groups (*p* < 0.007). Three-dimensional images of the surface roughness are shown in Figure 4. The 20% CP/CS and the 23% CP/CS groups showed more gross surface roughness than the others.

### 3.4. Mechanical Characterization

**Figure 5:**
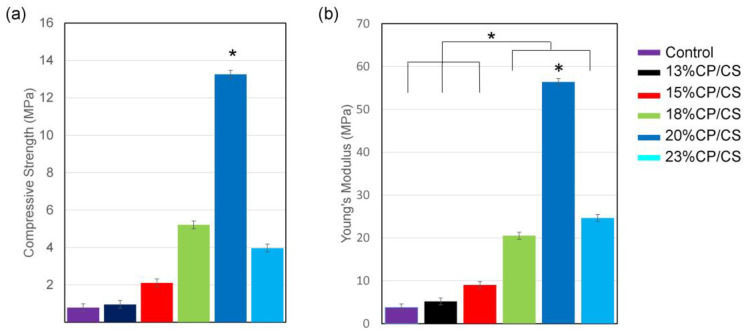
Mechanical characterization: (**a**) compressive strength at 80% strain and (**b**) Young’s modulus graph at 80% strain (* *p* < 0.05).

The 20% CP/CS group showed the highest compressive strength and highest Young’s modulus by a significant difference. In addition, Young’s modulus of the 18%, and 23% CP/CS groups significantly displayed more elasticity than the control, at 13% CP/CS, and the 15% CP/CS groups. Notably, increasing the percentage of CP/CS resulted in a rise in the compressive strength and of Young’s modulus, except for the 23% CP/CS (Figure 5). 

### 3.5. Swelling Property

**Figure 6:**
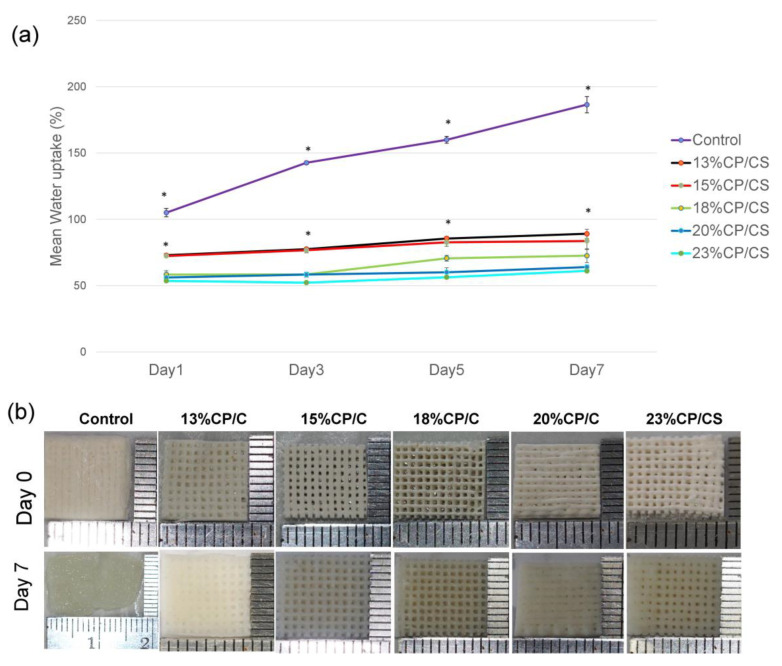
Swelling test: (**a**) percent values of water uptake after soaking in PBS (* *p* < 0.05), (**b**) swelling of the scaffolds after soaking in PBS at days 0 and 7.

The swelling ratio of the control group was statistically different than the other groups (*p* < 0.00). It rapidly increased until it reached 186 ± 6% on day 7, whereas the swelling ratios of the other experimental groups increased gradually. The 23% CP/CS group showed the lowest absorption (Figure 6a). The five experimental groups demonstrated no structural changes, but the control group was almost double in size on day 7 (Figure 6b).

### 3.6. Degradation Rate

**Figure 7:**
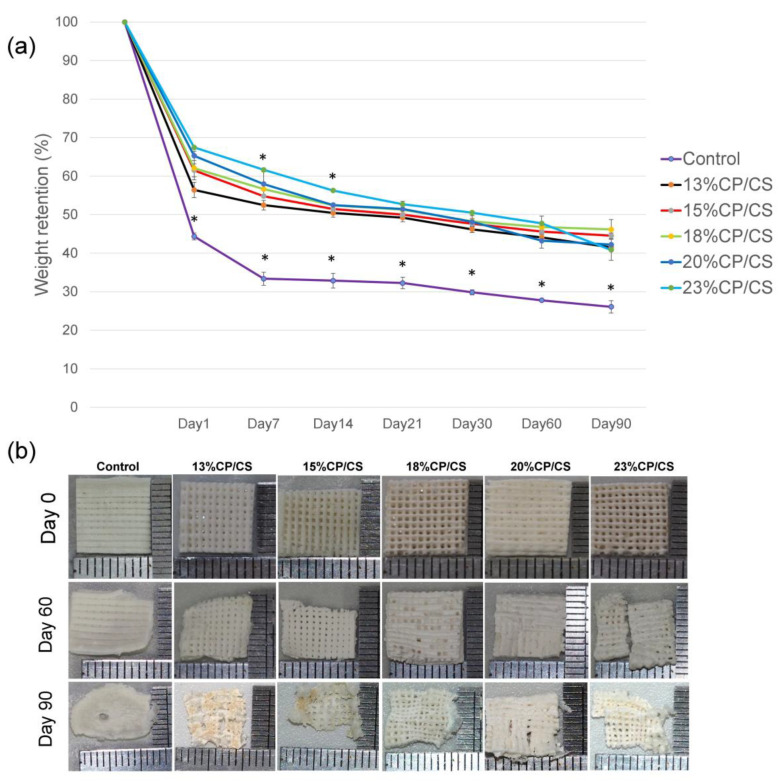
Degradation rate test: (**a**) percent of weight retention graph (* *p* < 0.05), (**b**) photos of scaffold degradation on days 0, 60, and 90.

The 23% CP/CS group showed the highest remaining weight, and the data were statistically significant on days 7 and 14 (*p* = 0.000). On the other hand, the control group statistically resorbed more than the others (*p* = 0.000) (Figure 7). The five experimental groups dimensionally retained the original shape until day 60; however, on day 60, the 23% CP/CS group cracked. All of the scaffolds changed irregularly in three months.

### 3.7. Cell Attachment and Spread

**Figure 8:**
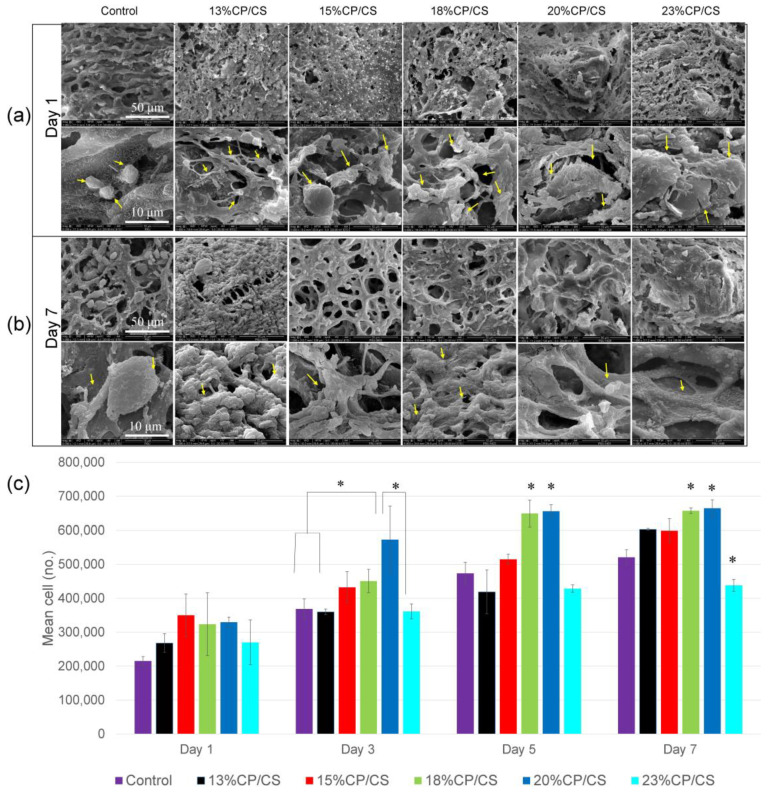
(**a**,**b**) SEM observed osteoblast cell attachment and spread on the surfaces of the scaffolds on days 1 and 7, yellow arrows indicate the osteoblast cells. (**c**) The cell proliferation rate was observed using PrestoBlue^®^ at an optical density of 600 nm at days 1, 3, 5, and 7 (* *p* < 0.05).

Osteoblastic attachment on the scaffold surface was observed by SEM. The control group showed the shortest pseudopodia and the lowest number of osteoblast cells at day 1. In comparison, the other groups presented attachment between the pseudopodia and material surface. Furthermore, the spread of osteoblast cells proliferated more in the experimental groups (Figure 8a). On day 7, the experimental groups had more cell spreading, and the cells nearly covered the entire surface of the material except for the control group, which demonstrated some non-attached osteoblast cells (Figure 8b).

### 3.8. Cell Viability

**Figure 9:**
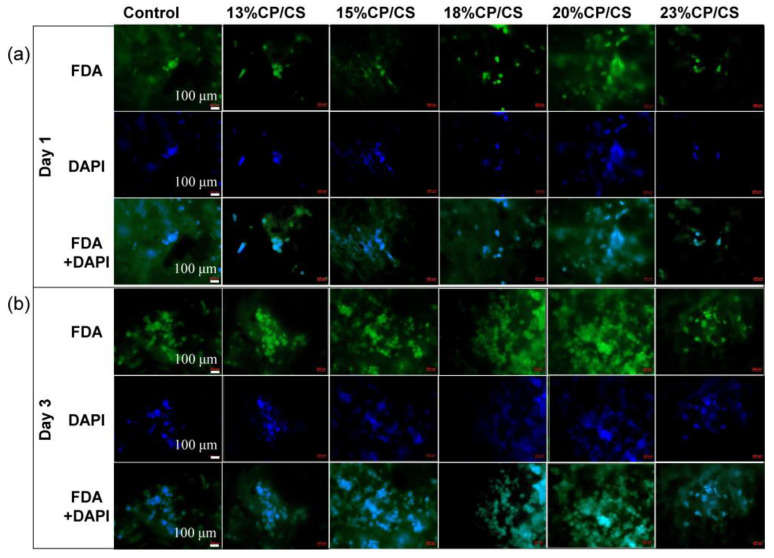
Fluorescence microscopy observed cell viability on the surface of the scaffolds on days 1 (**a**) and day 3. (**b**) The top rows show living cells detected by FDA in green. The middle rows show staining of nuclei in blue. The bottom rows show merged images of FDA + DAPI.

A confocal microscope was used to observe the green and blue fluorescence that were interpreted as being living cells. On day 1, living cells demonstrated a light green aura and light blue staining of the cell nuclei (Figure 9a). After three days, the viable cells presented with large nuclei, especially in the 15% CP/CS, 18% CP/CS, 20% CP/CS, and 23% CP/CS groups. Furthermore, the number of cells increased in all groups (Figure 9b).

### 3.9. Cell Proliferation

Optical density measured at 600 nm revealed that the mean number of osteoblast cells was approximately 55 × 10^4^ cells in the 20% CP/CS group, which had the highest cell proliferation that was statistically significant at day 3 (*p* < 0.003). Moreover, cell proliferation in the 18% CP/CS and 20% CP/CS groups showed statistical differences on days 5 and 7 with an average of 65 × 10^4^ cells (*p* < 0.000) (Figure 8c).

## 4. Discussion

Synthetic bio-resorbable composite materials are becoming increasingly important due to the availability of 3D printing to fabricate scaffolds. The combination of appropriate materials is the essential key for the 3D printing of scaffolds. Using CP/CS can increase the amount of calcium and phosphate ions to initiate osteoid formation [40]. At the same time, alginate/cellulose can be used to fabricate a flexible multilayered scaffold with a stable interconnected structure. Our study established a novel ratio of four materials to construct a suitable 3D morphology.

FDM is another technique for 3D printing that is generally used to extrude thermoplastic materials [41]; however, the filaments from FDM 3D printing are heated during the fabrication process. Since natural polymers are sensitive to heat, FDM was not preferred for this research. In addition, 3D printing should avoid filament breakage during printing.

The parameters of printing a 3D scaffold are important. If the speed or the pressure of the cartridge is too high, the result is a small filament. In addition, there are multiple types of nozzle tips available for printing. The 200 μm diameter nozzle tip was selected in this study to form the proper small filament. The compensated gel expansion method was performed prior to the completed gel set according to Markstedt et al. [42]. The 2.5–3.0% concentration of alginate was unable to obtain the architecture after printing.

It is easy to control the filament size and the spaces between each filament in 3D printing. The mean pore sizes of the fabricated scaffolds ranged from 298 to 377 μm with no significant difference. Nevertheless, the authors noticed that the cross-linking spray possibly caused variation in pore size during the initial reaction. Bagheri Saed et al. reported that 22–45% biphasic calcium phosphate (BCP) in 3D printing resulted in a pore range of 367–414 μm [43]; however, pore diameters of 300–400 μm promote proper cell proliferation [44] and are suitable for neo-vascularization [45]. The control group did not demonstrate cell proliferation because pores were not present in this group.

The filament pattern was staggered at 0° and 90° degrees, which provided more surface exposure for cell adhesion of the pre-osteoblast cells. In this study, the SEM images demonstrated a greater number of initial cell attachments at day 1 in the experimental groups than the control group. Furthermore, SEM at day 7 clearly showed the lowest cell accumulation in the control group (Figure 8b), which was due to the low viscosity of the gel during printing that led to fusion of the filaments. Lee et al. compared the staggered and normally aligned filaments. They reported that staggered filaments with a pore size of 350 μm allowed for a greater pre-osteoblast cell proliferation rate at days 1, 3, 7 because staggered filaments slowed the rate of cell movement during cell loading [46]. Moreover, Rotbaum et al. [47] stated that staggered filaments provided stress–strain resistance for a better stress distribution.

FTIR indicated optimal surface energy as an osteoconductive property resulting in surface roughness, swelling, degradation, initial cell attachment, and cell proliferation [48].

Surface roughness is one factor that affects cell adhesion, and a roughened surface often has a larger surface area. In addition, a report stated that loading with CP could be a benefit, by increasing the surface roughness to provide more binding sites for cell attachment [49]. An optimal roughness between 18–187 nm was reported for a proper cell proliferation rate [50]. In the current study, the surface roughness of the scaffolds was nearly smooth, especially in the control group, which was statistically significant. Nevertheless, the roughness increased in the other groups from the phosphate and carbonate groups at the surface. The roughness average ranged from 43 to 59 nm after adding the mixed CP/CS. This was similar to a study by Redey et al. that reported an increased surface roughness of about 24 nm after adding phosphate and carbonate. Moreover, they also reported high human osteoblast cell attachment on the phosphate and carbonate surface at 18 h, and cell amplification also increased continuously [48]; however, the results showed no correlation between the amount of CP/CS and roughness since the scaffold deformed during the freeze-drying process, which definitely affected needle touch perceptions [51].

This current study modified the weight percentage and base gel composition of Alg/cellulose based on a study by Schütz et al. which reported 0.02–0.08 MPa of compressive strength at 80% strain and 0.01–0.15 MPa of Young’s modulus [32]. The results of the control group in this study were a compressive stress of 0.80 MPa, and a Young’s modulus of 3.7 MPa; since this study used a different type of cellulose, which was HPMC, that was confirmed to improve the mechanical properties [52]. The compressive strength and Young’s modulus in this study increased as Alg/HPMC was reduced. In particular, the 20% CP/CS group showed the best mechanical properties. Nevertheless, when the CP/CS of the scaffold increased to 23%, the result was a brittle scaffold [38]. Moreover, the compressive strength and modulus of tetra-components in this study showed higher values compared with pure CP/Alg components [53]. The authors assumed that CS and HPMC enhanced the mechanical properties. Similarly, the 85% CS ink presented a higher flexural strength than 100% CS after soaking in the solution [54]. In addition, 22% BCP, hydroxyapatite (HA)/α-tricalcium phosphate (TCP) 3D printed scaffolds resisted compression and provided elasticity that was lower than the findings of this study (compressive strength 1.6 MPa, elasticity 34 MPa, and 2.6 MPa) [43,55]. 

When the scaffolds were soaked in PBS, the control group was the only group that showed a significant dimensional change because the carboxylic acids and the carboxylate ions of alginate probably increased the swelling ratio [56] in the 13% CP/CS and 15% CP/CS groups due to a significant absorption of water at 70–80%. Nevertheless, the CP particles played a vital role by preferentially absorbing water instead of the CS particles, which prevented excessive scaffold swelling. If CS is allowed to absorb water, the CS will transform from the hemihydrate form into the dehydrate form [38,57]. Swelling rates of 50–60% were observed in the 18% CP/CS, 20% CP/CS, and 23% CP/CS groups.

The CP prevented the CS from transforming into calcium sulfate dihydrate, which can be demonstrated by X-ray diffractometer spectra, since calcium sulfate dehydrate was not created [38]. In this study, the control group had a weight gain of more than 100 times due to the large amount of alginate. Not only did the alginate induce the swelling, but the hydroxyl groups also interacted with the water molecules. Although the 13% CP/CS had the lowest amount of CS, it demonstrated the greatest amount of water absorption compared with the other experimental groups. This was likely due to small clusters of CS that allowed the hydroxyl groups of the cellulose to be in contact with the water [57].

The degradation rates in the experimental groups in this study were similar to a study by Wu et al. [38] which tended to decrease by 30% to 40% at day 1, and remained approximately at 40%; however, the 23% CP/CS group showed structural losses due to the vast amounts of CP/CS. All experimental groups showed a degradation trend directly proportional to the percentage of water uptake efficiency and the water permeability of the scaffold. Similarly, the 45% BCP scaffold showed higher weight loss than the 22% BCP [43]. In addition, all experimental groups showed rapid weight loss on day 1, due to the early discharge of CS [38], which created poor crystalline structures as a result of the movement of the Ca and S ions into the water [58] that resulted in apatite formation [59]. The printing ink that contained 46% HA or β-TCP presented Ca and P release until day 14 [60]. In addition, all groups were capable of maintaining the structure until day 21 because the ratio of Alg to HPMC was nearly the same, which affected the cell behaviors [33]. The accumulative Ca ions that were released until day 21 resulted in the promotion of bone cell proliferation and differentiation into mature bone [33,61].

Cell behavior depended on the precipitation process of CP/CS. Calcium ions have a positive surface charge that can react with the negatively charged surface of the phosphate ions in human body fluids to form a poor Ca^2+^/ACP-like human bone apatite [62,63]. From our cell proliferation results, the 13% CP/CS, 15% CP/CS, 18% CP/CS, and 20% CP/CS groups promoted cell proliferation more than the control group. Nevertheless, the 23% CP/CS group was different because the amounts in the carbonate and phosphate groups were greater, which slowed cell proliferation [64]. Kilian et al. stated that the reaction between cross-linking and the Alg/HPMC base presented Ca ions to enhance living cell accumulation, which was similar to the control group [33]; however, the alginate reduced cell attachment [56]. Hence, SEM showed low cell attachment and a low cell accumulation rate in the control group. In contrast, less alginate could increase cell proliferation, especially in the 18% CP/CS and 20% CP/CS groups, which was significantly evidenced by the higher cell proliferation rates on days 5 and 7; however, the 23% CP/CS group was different after soaking in PBS for 7 days, which lost stability between the filaments. Nevertheless, the 22–45% BCP scaffolds presented lower cell proliferation than the positive control because the chemical chains broke into the culture medium [43].

None of the experimental groups revealed significant physiochemical properties; however, the mechanical properties of scaffolds in tissue engineering are crucial for cell culture and therapeutic applications. Cortical bone has a compressive modulus of 17–20 GPa and a compressive strength of 106–133 MPa. The compressive strength of cancellous bone is 2–12 MPa [65,66]. This study found that the compressive strength was less than 13 MPa, which was closer to cancellous bone; therefore, the strength of all scaffolds was adequate for non-load bearing applications in oral and maxillofacial bone tissue engineering. Trabecular bone compressive strength ranges from 0.22 to 10.44 MPa, whereas elastic modulus ranges from 3.5 to 125.6 MPa [67]; however, further research should be conducted to incorporate osteoblast cells into the bio-ink for bone tissue engineering.

## 5. Conclusions

Scaffolds from 3D printing using various amounts of CP/CS and alginate/cellulose were fabricated with suitable geometries and mimicked the extracellular matrix during initial osteoblast cell attachment and proliferation. All experimental groups had pore sizes in the range of 298–377 μm with staggered filaments. The surface roughness ranged from 43 to 62 nm, which promoted cell adhesion. The 20% CP/CS revealed the highest compressive strength and Young’s modulus, which was statistically significant. All experimental groups demonstrated more than 50% swelling. The 23% CP/CS groups cracked easily after soaking in PBS for two months. The 18% CP/CS and 20% CP/CS groups showed the most osteoblast cell proliferation at days 3, 5, and 7. The 20% CP/CS group was the most suitable for osteoblast cell attachment and proliferation.

## Figures and Tables

**Table 1:** Pore sizes and surface roughness averages of all groups.

Groups	Pore Size (μm), Surface	*p*-Value	Pore Size (Μm), Cross-Section	*p*-Value	Surface Roughness Average (nm)	*p*-Value
Control	0.00 ± 0.00	0.000	0.00 ± 0.00	0.000	9.02 ± 4.49	0.007
13% CP/CS	315.61 ± 42.31		334.00 ± 86.65		55.34 ± 4.66	
15% CP/CS	299.68 ± 48.92		377.09 ± 69.77		45.93 ± 7.46	
18% CP/CS	298.65 ± 55.09		323.96 ± 83.05		43.22 ± 4.89	
20% CP/CS	316.36 ± 52.95		316.60 ± 71.57		62.53 ± 9.43	
23% CP/CS	306.48 ± 48.14		327.99 ± 80.36		59.00 ± 5.06	

Data are presented as mean ± standard deviation.

## Data Availability

Not applicable.
